# Exploring potential mediating mechanisms between maladaptive perfectionism and athlete burnout based on multi-theory perspectives

**DOI:** 10.3389/fpsyg.2024.1416281

**Published:** 2024-09-13

**Authors:** Weiye Kang, Chen Gong

**Affiliations:** College of Physical Education, Northeast Electric Power University, Jilin, China

**Keywords:** maladaptive perfectionism, athlete burnout, fear of failure, self-handicapping, mediating role

## Abstract

**Background:**

Athletes with maladaptive perfectionism are vulnerable to experiencing a variety of psychological issues, such as burnout. Burnout in athletes can have detrimental effects on their performance and careers. The potential mechanisms by which fear of failure and self-handicapping explain the association between maladaptive perfectionism and athlete burnout remain understudied. This study examined their mediating role in the relationship between maladaptive perfectionism and athlete burnout.

**Methods:**

A total of 221 athletes were chosen to participate in a cross-sectional survey study. Data analysis was carried out using SPSS and AMOS structural equation modeling. The participants filled out self-report assessments on maladaptive perfectionism, fear of failure, self-handicapping, and athlete burnout.

**Results:**

Analyses indicated that maladaptive perfectionism positively predicts fear of failure, self-handicapping, and athlete burnout. Fear of failure positively predicts self-handicapping and athlete burnout, while self-handicapping also predicts athlete burnout. In addition to the direct pathway, we identified three mediating pathways through mediation analyses: (a) an independent mediation of fear of failure (b) an independent mediation of self-handicapping (c) a chained mediation of both.

**Discussion:**

The results of this study provide a better understanding of the underlying mechanisms between maladaptive perfectionism and athletes burnout by considering fear of failure and self-handicapping as mediating variable factors. It is shown that the relationship between maladaptive perfectionism and athlete burnout can be partially explained through the mediating role of individuals’ fear of failure as well as self-handicapping behaviors. These insights offer a valuable foundation for the design of psychological interventions to address athlete burnout, enabling coaches and sport psychologists to develop more effective coping strategies for enhancing athletes’ psychological well-being and performance.

## 1 Introduction

Smith’s cognitive-emotional stress model (Smith, 1986) posits that athlete burnout results from a chronic imbalance between environmental demands and personal resources, representing the final stage of an athlete’s prolonged exposure to stressful situations. Athletes are increasingly acknowledged for their societal influence and often face psychological stressors in rigorous training settings, which potentially leads to mental health issues (Pascoe et al., 2022) such as athlete burnout (Li et al., 2022). Burnout was originally introduced by Maslach and Jackson (1984) to describe the exhaustion of psychological resources in high-pressure work environments. While widely used, this concept is mainly associated with person-work scenarios and is not typically applied to sports, where athletes prioritize their athletic performance. Therefore, Raedeke (1997) introduced athlete burnout to explain the exhaustion of psychological resources in long-term and high-stress athletic environments. Three dimensions are included—(a) emotional/physical exhaustion, a negative reaction to the strong need for athletic training and competition; (b) sports devaluation, a loss of interest and desire to participate in sports; (c) a decline in athletic performance, a decrease in the sense of accomplishment of athletic skills and abilities.

Athlete burnout is becoming more prevalent (Madigan et al., 2022), with rates ranging from 1 to 5% (Cresswell and Eklund, 2007). Athlete burnout comes with various negative outcomes, e.g., decreased motivation (Cresswell and Eklund, 2005), poor athletic performance (Jowett et al., 2016), strained interpersonal relationships (Choi et al., 2020), and increased risk in sports (Seehusen et al., 2023). Over the last three decades, researchers have delved into the mechanisms of athlete burnout (Smith et al., 1992) and explored the factors that contribute to athletes being more prone to burnout. Strategies can be implemented by identifying and addressing these factors to mitigate athlete burnout.

Previous studies have made several attempts to describe and explain athlete burnout. The cognitive-emotional stress model (Smith, 1986) proposed by Smith has been widely cited to explain the causes of burnout. The model postulates burnout as a four-stage process in which stress and burnout develop in parallel. In the first stage, athletes are subjected to demands such as high training loads, extreme expectations, and parental pressure. The second stage involves a cognitive assessment of the current situation. When an athlete views the situation as more daunting than others and experiences helplessness, burnout symptoms typically emerge in the third stage. These symptoms include tension, fatigue, and insomnia, arising when the demands are perceived as overwhelming or threatening. Finally, the physiological response will result in decreased performance, avoidance behaviors, and even withdrawal from the activity in stage four.

Athlete burnout is affected by both endogenous and exogenous factors (Guo et al., 2022). Maladaptive perfectionism, an internal factor within the individual athlete (Waleriańczyk and Stolarski, 2022), can contribute to burnout through endogenous factors such as perceived stress (Olsson et al., 2022). Not limited to perfectionism, fear of failure has also been shown to be highly correlated with athlete burnout (Yıldırım et al., 2023). According to Smith’s cognitive-emotional stress model (Smith, 1986), stressors (self-handicapping), which are potential factors for burnout, may serve as explanatory factors to enrich the burnout mechanism.

Perfectionism as a personality trait is characterized by the constant pursuit of flawlessness and setting extremely high standards of performance. It is accompanied by a propensity to critique one’s behavior with excessive severity (Flett and Hewitt, 2002). Frost et al. (1990) conceptualized perfectionism as a multidimensional trait that has been widely cited. Perfectionism has both adaptive and maladaptive dimensions. The more maladaptive qualities there are, the greater the perceived stress (Hamachek, 1978). When perfectionism is examined within the realm of sports, it is vital to differentiate between adaptive and maladaptive perfectionism. These two dimensions display divergent patterns of correlation with a variety of outcomes. Adaptive perfectionism, marked by an individual’s drive to exceed expectations (i.e., perfectionist efforts), contrasts with maladaptive perfectionism, which is linked to a fear of being assessed or judged by others (Hill and Curran, 2015; Enns et al., 2001). Both adaptive perfectionism and maladaptive perfectionism are correlated with athlete burnout (Garinger et al., 2018), with the former negatively predicting burnout and the latter positively predicting burnout (Hill and Curran, 2015), and with stress mediating this relationship to some extent (Garinger et al., 2018). This coincides with the cognitive-emotional stress model.

Perfectionism, being a relatively stable trait, necessitates the identification of mediating variables that can moderate the relationship between maladaptive perfectionism and athlete burnout. This understanding significantly enhances intervention programs for addressing athlete burnout. Notably, fear of failure and self-handicapping, as potential stressors, demonstrate a significant correlation with perfectionism and athlete burnout.

Fear of failure initially emerges in competitive sports environments, rooted in an athlete’s difficulty in effectively managing and controlling specific athletic situations (Conroy and Elliot, 2004). This can lead to a bias towards the pursuit of achievement-avoidance goals, causing the athlete to experience heightened anxiety. Consequently, a range of consequences occur, e.g., feelings of shame, diminished self-esteem, and apprehension about an uncertain future (Murcia and Marín, 2011). Considering that most of the athletic behaviors of young athletes are regularly assessed based on success criteria (Sagar et al., 2007), the self-perception of the athletic experience tends to be a fear of making mistakes. Fear of failure occurs when athletes are afraid of not being recognized (González-Hernández et al., 2021). Conceptually, fear of failure is intertwined with the perceived criticalness of oneself or others. It shares a common emphasis on avoiding perceptions of incompetence, akin to certain facets of perfectionism (Zhang et al., 2022). Avoidance and reduced achievement, associated with the fear of failure, are linked to self-handicapping (Török et al., 2022) and burnout, respectively (Waleriańczyk and Stolarski, 2022).

Athletes complaining about injuries and lifting others during training and before competitions are common examples of self-handicapping in sports. Berglas first introduced and defined self-handicapping as an individual’s avoidance or mitigation of being negatively affected by failure. Taking action or changing goals is used as an external excuse to explain away expected failure (Covington, 1993). Later Carolyn defined self-hindering as a self-protective attributional strategy in which individuals intentionally put resistance to their success (Murray and Warden, 1992). There are few studies on self-handicapping in athletes. The process of self-hindering should be thoroughly understood because of its negative impact on sports performance and athletes’ psychology (Schwinger et al., 2014).

Perfectionism and athlete burnout have been studied for a long time. In contrast to positive adaptive perfectionism, maladaptive perfectionists experience negative aspects of imposing excessively high demands on both themselves and others (Flett and Hewitt, 2005). Maladaptive perfectionism, exhibiting a moderate to strong positive correlation with athletic burnout (Hill and Curran, 2015), is a significant positive predictor of athlete burnout (Garinger et al., 2018). Fear of failure can be categorized as part of a negative emotion or emotional distress from concerns about future uncertainty, doubts about one’s abilities, or fear of the failure consequences. The tendency for individuals to prioritize the avoidance of achievement goals is a notable characteristic of those with fear of failure. This trait is strongly associated with the manifestation of burnout symptoms (Waleriańczyk and Stolarski, 2022).

Fear of failure has a significantly positive correlation with the reduced achievement of athlete burnout (Gustafsson et al., 2016). Achievement motivation theory (AMT) (Cury et al., 2006) suggests that fear of failure may lead to avoidance of challenges and competition, which affects an individual’s motivation for achievement. According to AMT, an individual’s achievement motivation affects their response to failure. When athletes are afraid of failure, they may feel anxious, nervous, or even depressed, which can lead to burnout and negative behaviors.

Research on the identified variables related to self-handicapping focuses on lowered self-esteem, social anxiety (Strube, 1986), maladaptive perfectionism (Hobden and Pliner, 1995), and lower achievement (García, 1995) which also serve as one dimension of burnout. Self-handicapping strategies may protect self-worth in the short term but are highly likely to lead to burnout in the long term (Akın, 2012). Self-handicapping is positively correlated with burnout. Slade and Owens (1998) proposed that maladaptive perfectionism is based on negative reinforcement and fear of failure. Zhang et al. (2022) demonstrated a positive correlation between maladaptive perfectionism and fear of failure. Athletes’ attributions about sports outcomes affect their emotions, decisions, expectations, and behaviors (Malle, 2004). Several studies based on attribution theory (AT) have shown that attributions are related to specific components of personality, including trait anxiety (Khodayarifard et al., 2006), trait self-handicapping (Greenlees et al., 2006), and perfectionism (Stoeber and Becker, 2008).

Perfectionists tend to have very high expectations of themselves and others; they strive for perfection and are demanding of themselves. When confronted with failures or errors, perfectionists with fear of failure often attribute responsibility to internal, stable factors that are specific to themselves, such as “I did not work hard enough” or “I was not smart enough.” This type of attribution can lead to frustration, anxiety, and even fear of failure because they are afraid that they will not be able to live up to the standard of perfection. Self-handicapping, a strategy linked to attribution (Rhodewalt, 2008; Higgins et al., 1990), is conceptualized as a maladaptive coping mechanism. It has a significantly positive correlation with maladaptive perfectionism (Slade and Owens, 1998; Török et al., 2022). Athletes with high levels of maladaptive perfectionism may experience greater fear of failure and tend to engage in self-handicapping to avoid failure. This can manifest as athlete burnout in turn. That is, fear of failure and self-handicapping may be mediating variables between perfectionism and athlete burnout. However, previous studies have paid little attention to the relationship between these four variables.

**Figure 1:**
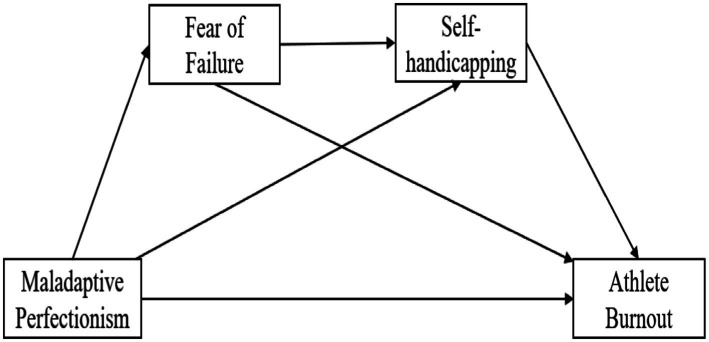
Hypothesized model.

The work aimed to determine the relationship among perfectionism, fear of failure, self-handicapping, and athlete burnout. Based on the literature review mentioned above, a structural model was proposed (Figure 1). Based on this model, four main hypotheses were proposed.

*H1*: Maladaptive perfectionism positively predicts athlete burnout.

*H2*: Fear of failure and self-handicapping positively predict athlete burnout.

*H3*: Fear of failure mediates the relationship between maladaptive perfectionism and athlete burnout.

*H4*: Self-handicapping mediates the relationship between maladaptive perfectionism and athlete burnout.

*H5*: Fear of failure and self-handicapping play a chain mediating role between maladaptive perfectionism and athlete burnout.

## 2 Materials and methods

### 2.1 Participants

A convenience sampling method was used to complete the questionnaire with 230 athletes from competitive team (e.g., volleyball, basketball) and individual sports (e.g., track and field, cycling) in Jilin Province, China. Nine participants were excluded from the data analysis due to extreme values and missing values (Gröschke et al., 2022). The effective response rate was 96.1%. Based on Monte Carlo simulations, the work calculated the required sample size for the relevant models and inverted the current effect sizes of the chain mediation model. The minimum sample size for the chained mediation model between maladaptive perfectionism and athlete burnout, with a power value of 0.8, was found to be *N* = 126. With a power value of 1.0, required sample size *N* = 253. The current dataset’s subject size aligned with the predicted sample size. Furthermore, the effect values of the reversed model indicated that our sample size (*n* = 221) (with *α* = 0.96) was adequate for indirect effects analyses (Schoemann et al., 2017). The questionnaire consisted of participant consent, demographics, and a research instrument section. Of the 221 participant responses analyzed, 136 (61.5%) were male athletes and 85 (38.5%) were female athletes. Table 1 lists specific information.

**Table 1:** Basic information of respondents (*n* = 221).

Characteristics		Frequency (*n*)	Effective percentage	Cumulative percentage
Gender	Male	136	61.5	61.5
	Female	85	38.5	100.0
	(Total)	221	100.0	
Age	17 years old and under	53	24.0	24.0
	18–21 years old	81	36.6	60.6
	22 years old and above	87	39.4	100.0
	(Total)	221	100.0	
Training years	4 years and less	105	47.5	47.5
	4–8 years	74	33.5	81.0
	9 years and more	42	19.0	100.0
	(Total)	221	100.0	

Data collection was conducted after requesting the cooperation of participants and by obtaining consent from the person in charge (coach) of the sports team. We personally visited each sports team and explained the method for completing the questionnaire, and any precautions for athletes. Before the initiation of data collection, all participants were advised of their voluntary involvement, the conditions of anonymity, and their entitlement to withdraw from the work at any point during the data collection process.

### 2.2 Methods

Missing data and distribution analyses were conducted to examine the entire data set before data analysis. Then, validity and reliability tests were performed on all study data. A confirmatory factor analysis (CFA) was conducted to assess the construct validity of all scales. This approach replaced EFA because the factorization of all instruments was well-established and widely reported. Therefore, there was no need to explore underlying factors. Structural validity was assessed based on model-fitting indices such as the *χ*^2^/df statistic, comparative fit index (CFI), adjusted goodness of fit index (AGFI), Tucker-Lewis index (TLI), root mean square error of approximation (RMSEA) and standardized root mean square residual (SRMR). All results were analyzed using the statistical package for social sciences (SPSS) and the structural equation modeling of AMOS.

#### 2.2.1 Independent variable

Maladaptive perfectionism was measured using the *Perfectionism in Sport Scale* (*MPS-S-C*) revised by Lian et al. (2007). The original scale was the English version of the *MPS-S* developed by Dunn et al. (2002). The *MPS-S-C*, revised by Lian through in-depth multiple measurements of Chinese athletes, contained 37 items and 5 dimensions. Personal standards belonged to adaptive perfectionism, and Cronbach’s *α* = 0.969, indicating ideal internal consistency. Maladaptive perfectionism included repetitive thinking (RTM), concern for mistakes (CM), perceived parental stress (PPS), and perceived coaching stress (PCS). Higher scores indicated a greater tendency toward maladaptive perfectionism. Cronbach’s *α* was 0.886 for the total questionnaire of maladaptive perfectionism, with revised χ^2^/df = 2.077, GFI = 0.914, AGFI = 0.878, CFI = 0.959, TLI = 0.949, RMSEA = 0.07, and SRMR = 0.054.

#### 2.2.2 Dependent variable

The A*thlete Burnout Questionnaire* (*ABQ*) was developed by Raedeke and Smith (2001), and the questionnaire revised by Liwei and Zhi (2010) in China was adopted. The questionnaire consisted of 15 questions with three dimensions: emotional/physical exhaustion, reduced accomplishment, and negative evaluation of exercise. Higher scores represented higher levels of burnout. Cronbach’s *α* was 0.795 for the total questionnaire, with revised χ2/df = 3.037, GFI = 0.953, AGFI = 0.898, CFI = 0.965, TLI = 0.944, RMSEA = 0.096, and SRMR = 0.0484.

#### 2.2.3 Mediation variable (i)

The Chinese version of the *Performance Failure Assessment Inventory* (*PFAI*) revised by Cho and Lu (2005) was adopted. The scale consisted of 18 items with 4 dimensions (i.e., fear of facing humiliation and embarrassment, fear of lowered self-esteem, fear of losing the interest of significant others, and fear of being criticized by significant others). Higher scores indicated higher levels of fear of failure. Cronbach’s *α* was 0.896 for the total questionnaire. Since the *PFAI* contained many items, the work re-categorized 18 items of the *PFAI* based on the item-packaging theory to simplify the measurement structure (Wu and Wen, 2011). The items were categorized into four item packages, which served as the observables of the *PFAI*. Revised χ^2^/df = 3.212, GFI = 0.986, AGFI = 0.928, CFI = 0.991, TLI = 0.972, RMSEA = 0.100, and SRMR = 0.0190.

#### 2.2.4 Mediation variable (ii)

The *Sports Self-Handicapping Scale* revised by Qing and Liwei (2008) was adopted. The scale contained 10 items and 1 dimension, for example, “When my athletic performance fails to meet the expectations of others, I will find some reasonable reasons to explain it.” Cronbach’s *α* was 0.910 for the total questionnaire, with χ^2^/df = 2.227, GFI = 0.952, AGFI = 0.913, CFI = 0.976, TLI = 0.967, RMSEA = 0.075, and SRMR = 0.0308.

### 2.3 Data analysis

SPSS 26.0 was used for data cleaning, common method bias, descriptive statistics, and correlation analysis. Based on the results and hypotheses of the above correlation analyses, a chain mediation model was established to validate the relationship between maladaptive perfectionism and athlete burnout. AMOS 28.0 was utilized to validate the fear of failure and self-limiting chained-mediated pathways. The work applied maximum likelihood (ML) and set the Bootstrap to resample 5,000 times for model estimation, which calculated 95% of confidence intervals.

## 3 Result

### 3.1 Test of common method biases

All items of the four key variables of the work were analyzed using the Harman one-way test (Podsakoff et al., 2003) to test common methodological bias. The amount of variances explained by the first factor was 32.57%, which was lower than 40%. The common methodological bias did not have a significant effect.

### 3.2 Descriptive statistics

Table 2 lists the descriptive statistics (i.e., means and standard deviations), bivariate correlation coefficients, and internal consistency reliability estimates for the main variables. All the variables have acceptably good internal consistency reliability. Correlation analyses show that adaptive perfectionism is not significantly correlated with maladaptive perfectionism (*r* = 0.126 and *p* > 0.05), fear of failure (*r* = 0.053 and *p* > 0.05), self-handicapping (*r* = −0.038 and *p* > 0.05), and athlete burnout (*r* = −0.005 and *p* > 0.05). Maladaptive perfectionism is positively correlated with fear of failure (*r* = 0.532 and *p* < 0.001), self-handicapping (*r* = 0.576 and *p* < 0.001), and athlete burnout (*r* = 0.431 and *p* < 0.001). Besides, fear of failure (*r* = 0.668 and *p* < 0.001) and self-handicapping (*r* = 0.503 and *p* < 0.001) are positively associated with athlete burnout. These results provide preliminary support for hypothesized chain mediation.

**Table 2:** Correlation analysis.

	Descriptive Statistics			Correlation				
	M	SD	α	1	2	3	4	5
Adaptive Perfectionism	3.60	0.74	0.547	1				
Maladaptive Perfectionism	3.00	0.68	0.886	0.126	1			
Fear of failure	2.87	0.99	0.896	0.053	0.532***	1		
Self-handicapping	2.60	0.66	0.910	−0.038	0.576***	0.347***	1	
Athlete burnout	2.51	0.68	0.795	−0.005	0.431***	0.668***	0.503***	1

***p < 0.001.

### 3.3 Mediating effect test

**Figure 2:**
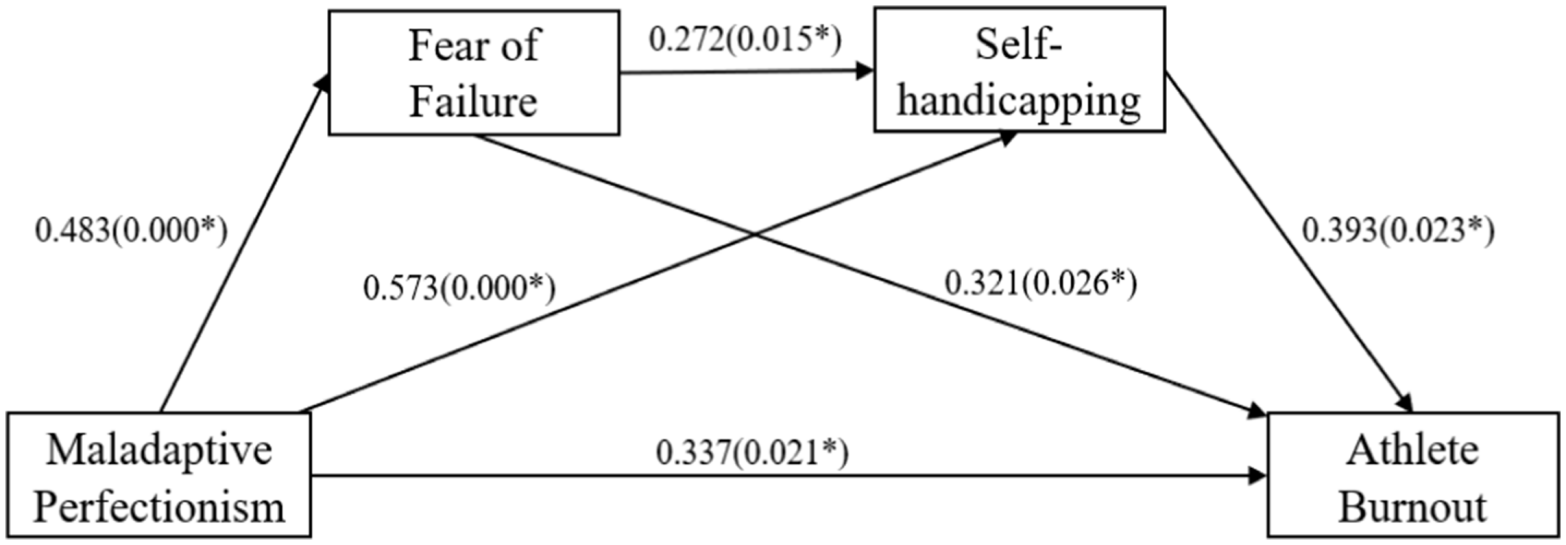
The mediating effect model of fear of failure and self-handicapping on the relationship between maladaptive perfectionism and athlete burnout.

Mediation analysis was conducted using the path analysis using AMOS version 28. The Bootstrap method was adopted to analyze the mediating effect, with maladaptive perfectionism as the independent variable, fear of failure and self-handicapping as the mediating variable, and athlete burnout as the dependent variable. Specifically, AMOS was used to construct bias-corrected, bootstrapped confidence intervals with 5,000 iterations to test these two hypotheses involving indirect effects. The indirect effect is significant if 95% of the confidence interval (CI) excludes zero. Figure 2 and Table 3 present the results. Structural equation modeling of maladaptive perfectionism on athlete burnout was established. The total effect of maladaptive perfectionism on athlete burnout was 0.395 (*p* < 0.001); the direct effect was 0.173; the indirect effect was 0.222; the coefficients were all significant. χ^2^/DF = 2.003, GFI = 0.857, AGFI = 0.820, CFI = 0.927, TLI = 0.916, and RMSEA = 0.068, indicating that the model is acceptable.

**Table 3:** Mediating effect test.

	β	Sig	LLCI	ULCI
Direct effects paths				
MP → FF	0.483	0.000	0.309	0.645
MP → SH	0.573	0.000	0.316	0.785
MP → AB	0.337	0.021	0.070	0.655
FF → SH	0.272	0.015	0.060	0.452
FF → AB	0.321	0.026	0.047	0.604
SH → AB	0.393	0.023	0.075	0.669
Effect type	Estimate			
Total	0.395	0.000	0.215	0.747
Direct	0.173	0.000	0.034	0.457
Indirect	0.222	0.000	0.114	0.428
Ind1 (MP → FF → AB)	0.079	0.018	0.014	0.198
Ind2 (MP → SH → AB)	0.116	0.007	0.043	0.242
Ind3 (MP → FF → SH → AB)	0.027	0.014	0.005	0.098

MP, maladaptive perfectionism; AB, athlete burnout; FF, fear of failure; SH, self-handicapping.

Specifically, maladaptive perfectionism positively predicted fear of failure (*β* = 0.483 and *p* < 0.001), self-handicapping (*β* = 0.573 and *p* < 0.001), and athlete burnout (*β* = 0.337 and *p* < 0.05); thus, hypothesis 1 was supported. Fear of failure (*β* = 0.321 and *p* < 0.05) and self-handicapping (*β* = 0.393 and *p* < 0.05) positively predicted athlete burnout; therefore, hypothesis 2 was supported.

Maladaptive perfectionism was found to positively predict athlete burnout through two indirect pathways: fear of failure (with indirect effect ind1 = 0.079; 95 CI [0.014, 0.198]) and self-handicapping (with indirect effect ind2 = 0.116; 95 CI [0.043, 0.242]). This supports hypotheses 3 and 4. Maladaptive perfectionism positively predicted athlete burnout through the chained mediation pathway (indirect effect ind3 = 0.027; 95 CI [0.005, 0.098]) with fear of failure and self-handicapping as the mediating variables. Therefore, hypothesis 5 was supported.

## 4 Discussion

Drawing on AMT and AT, the work constructed a chain-mediated model to synthesize the mechanisms linking maladaptive perfectionism, fear of failure, self-handicapping, and athlete burnout. The results indicated a positive predictive role of maladaptive perfectionism on athlete burnout as well as independent and chain-mediated roles of fear of failure and self-handicapping. Thus, one direct pathway and three mediating pathways are revealed, suggesting that athletes characterized by high maladaptive perfectionism come with higher levels of fear of failure and a higher propensity for self-handicapping, and appears the risk of athlete burnout. Athletes with high levels of maladaptive perfectionism are more likely to experience fear of failure and have a greater tendency toward self-handicapping, which poses a risk for athlete burnout.

### 4.1 Relationship between maladaptive perfectionism and athlete burnout

The correlation analysis and structural equation modeling suggest that maladaptive perfectionism is a crucial factor in an athlete’s psychological state (Crowell and Madigan, 2021). Excessively high standards and an excessive focus on mistakes positively predict stress and anxiety in athletes. The prolonged accumulation of these psychological burdens may result in psychological exhaustion and emotional dysregulation (Dunn et al., 2019). Also, maladaptive perfectionism can predict burnout by affecting athletes’ cognitive and emotional processes (one dimension of athlete burnout). Negative self-talk and excessive anxiety are shown to contribute to athletes’ negative feelings about training and competition, reduce their motivation and enthusiasm to participate in their sports, and exacerbate the process of developing burnout.

Correlation analysis indicates that adaptive perfectionism is negatively but not significantly associated with athlete burnout. This does not fit with some early findings (Madigan et al., 2016; Jowett et al., 2016). While adaptive perfectionism is typically viewed as being significantly negatively associated with burnout, the adaptive aspect of perfectionism remains highly contentious and distinctly context-dependent. Its impact on athlete burnout may vary depending on the environment and specific context (Smith et al., 2016; Smith et al., 2018). According to self-determination theory (SDT), an individual’s behavioral motivation is affected by three basic psychological needs, namely autonomy, sense of competence, and interpersonal relationships (Fan et al., 2023). Maladaptive perfectionism undermines the satisfaction of these basic needs, which causes athletes to lose a sense of control over their behavior and motivation to participate in the exercise process. Thus, burnout is exacerbated.

### 4.2 Mediating role of fear of failure and self-handicapping

Maladaptive perfectionism can indirectly affect athlete burnout through fear of failure and self-handicapping, acting as individual and serial mediators. Fear of failure and self-handicapping are important pathways through which maladaptive perfectionism positively predicts athlete burnout. They also represent the salient results of the work. Previous studies have not demonstrated this mediating role (Olsson et al., 2022; Jowett et al., 2021), and the findings of the work provide new perspectives on the influence of maladaptive perfectionism on athlete burnout.

Maladaptive perfectionism may trigger an individual’s fear of failure and criticism. Excessive focus on mistakes and repetitive thinking can cause individuals to become overly anxious and worried about failure. Perceived pressure from parents and coaches can also increase fear of failure (Sagar and Stoeber, 2009). This fear comes not only from external pressures and expectations as well as the individual’s internal questioning of self-competence and worth, which creates a rejection of failure and criticism (Gustafsson et al., 2016). Thus, fear of failure plays an important mediating role between maladaptive perfectionism and athlete burnout. This is one of the mechanisms through which maladaptive perfectionism impacts athlete burnout, serving as an internal driver of athlete burnout.

Maladaptive perfectionists often set excessively high expectations for themselves and frequently focus on mistakes and failures. This tendency may result in individuals questioning their abilities and worth and heightens the propensity for self-handicapping. Self-handicapping manifests in increased negative beliefs, decreased self-evaluation, and heightened negative emotions (Török et al., 2022; Coudevylle et al., 2011). These psychological effects further undermine individuals’ self-motivation and mental toughness, which makes them more susceptible to burnout. For instance, maladaptive perfectionists might overly concentrate on their errors during exercise, which declines confidence in their abilities. This is often paired with a greater inclination toward self-handicapping, and elevate the occurrence of negative emotions with athlete burnout. Thus, maladaptive perfectionism may positively predict athlete burnout through self-handicapping.

Drawing on the discovery that fear of failure and self-handicapping serve as chain mediators, the self-handicapping behavior in athletes can be influenced by fear of failure. This aligns with the findings of Pei (2013). Athletes who pursue perfection are prone to excessive pre-competition anxiety, excessive focus on mistakes and self-blame during the competition, and a sense of anxiety about failure (Anshel and Eom, 2003). Maladaptive perfectionists with irrational beliefs are more likely to experience fear of failure (Jing and He, 2010). When individuals have a high level of fear of failure, they are more inclined to engage in self-handicapping behaviors. Furthermore, athletes with lower self-efficacy are more prone to using self-handicapping strategies, and fear of failure has been observed to undermine self-efficacy (Longbottom et al., 2010). If they fail, they have to face embarrassment, shame, and other people’s criticism as well as lose other people’s trust and interest in themselves. This is accompanied by a reduced sense of achievement and emotional exhaustion. Consequently, the greater the concern about failure, the more likely individuals are to seek excuses to alleviate their stress, which fosters a tendency toward self-handicapping and experiences athlete burnout.

The following results can be obtained. First, we provided new perspectives on the mechanisms affecting athlete burnout by revealing the positive correlations among maladaptive perfectionism, fear of failure, self-handicapping, and athletic burnout. Second, the prior role of fear of failure in athletes’ self-handicapping (i.e., the positive effect of fear of failure on self-handicapping) suggested that the relationship should be taken into account. Third, although previous research has confirmed that maladaptive perfectionism predicted athlete burnout, the work studied the mechanisms affecting athlete burnout. Compared to previous findings, maladaptive perfectionism not only directly predicted athlete burnout, but also indirectly affected athlete burnout through the independent mediating and chain mediating effects of fear of failure and self-handicapping. The maladaptive perfectionism’s influence on athlete burnout was explored from a psychological perspective, which enriched the theoretical framework of the relationship between perfectionism and athletes’ psychological states. In particular, examining the mediating roles of fear of failure and self-handicapping has enhanced our understanding of the pathways through which maladaptive perfectionism operated. Results can offer new perspectives for future related research.

Our findings have important practical implications for enhancing athletes’ mental health and facilitating athlete burnout interventions. First, based on the fact that maladaptive perfectionism can positively predict athlete burnout, personality trait-based interventions can prevent athlete burnout. Since perfectionism is assumed to be constructed during the socialization process and is considered to be stable in adults as an enduring personality trait (Flett et al., 2011), coaches and mental health counselors should focus on younger athletes and their social environments (e.g., schools). This period is a key stage in the development of perfectionist traits (Melero et al., 2020). Second, the mediating role of fear of failure and self-handicapping in the relationship between maladaptive perfectionism and athlete burnout offers crucial insights into strategies that coaches can employ to assist athletes in developing a healthier perception of dealing with failure and frustration. For instance, coaches can utilize cognitive behavioral therapy (Barlow et al., 2004; Egan et al., 2014) to help athletes reframe their perception of failure, which highlights it as an opportunity for learning and growth and offers suitable support and encouragement (Gustafsson et al., 2014). Athletes can recognize the negative effects of self-handicapping behaviors and learn to cope with them through mental skill training and cognitive restructuring.

### 4.3 Limitations

There are three limitations to the work. Firstly, a cross-sectional quantitative design is employed to reveal correlations instead of establishing causation. Although maladaptive perfectionism leads to athlete burnout (Madigan et al., 2016), its relationship with mediating variables has not been longitudinally examined. Causal inference requires time-dimensional follow-up data to detect the role of the mediating variable in the causal chain. Its actual mediating effect cannot be confirmed through cross-sectional data alone (Květon et al., 2021). Therefore, it is not appropriate to draw any firm conclusions about the causal relationships among these variables. Future research employing longitudinal or experimental designs can be undertaken to confirm the causal relationships between the variables, which leads to more robust conclusions. Second, since all questionnaire responses are based on the athletes’ self-reported forms, the results may be affected by endogeneity bias. Thirdly, the sample size is limited as the participants are recruited from Jilin, China, which makes it challenging to generalize the findings to all athletes across China. Future studies should include more convincing samples to better support the current findings.

## Data Availability

The original contributions presented in the study are included in the article/supplementary material, further inquiries can be directed to the corresponding author.
